# Subdural hematoma, a rare complication of plasmodium falciparum malaria: A case report

**DOI:** 10.1016/j.ijscr.2024.110739

**Published:** 2024-12-13

**Authors:** Irene Kilenzi, Adil Datoo, Jacqueline Gabone, Elisamia Ngowi, Mugisha Clement Mazoko

**Affiliations:** a Department of Surgery, Aga Khan Hospital, P.O. Box 2289, Dar Es Salaam, Tanzania; b Department of Surgery, Aga Khan University, P.O. Box 38129, Dar Es Salaam, Tanzania; c Department of Paediatrics and Child Health, Aga Khan University, P.O. Box 38129, Dar Es Salaam, Tanzania; d Department of Radiology, Aga Khan Hospital, P.O. Box 2289, Dar Es Salaam, Tanzania

**Keywords:** Plasmodium falciparum, Malaria, Subdural hematoma, Case report

## Abstract

**Introduction:**

Malaria is a vector-borne disease caused by protozoa and a major cause of mortality and morbidity worldwide. Falciparum malaria causes most malaria-related deaths, and rarely is it associated with subdural hematoma.

**Case presentation:**

We present a 40-year-old male diagnosed with falciparum malaria. The patient was on treatment for malaria when he developed neurological symptoms, and a CT scan showed subdural hematoma necessitating surgical intervention.

**Discussion:**

This case highlights subdural hematoma as a very rare complication of falciparum malaria. There was a recurrence of the subdural hematoma within 2 weeks despite initial intervention, but the patient attained resolution of symptoms after re-evacuation.

**Conclusion:**

Subdural hematoma is a very rare complication of plasmodium falciparum and physicians ought to have a high index of suspicion especially in endemic regions.

## Highlights

• Plasmodium Falciparum is associated with most deaths caused by malaria and severe complications.
• Sub-dural hematoma is an infrequent complication of plasmodium falciparum malaria.
• Prompt neuro imaging ensures early evacuation of hematoma and quicker recovery.
• Recurrence of the subdural hematoma is possible even after resolution of malaria infection.

## Abbreviations

CT SCAN: Computed Tomography
MRI: Magnetic Resonance Imaging
AVM: Arterial Venous Malformation
WHO: World Health Organization
PCR: Polymerase Chain Reaction

## 1 Introduction

Malaria is a deadly disease transmitted by mosquitoes and caused by the protozoan parasites: Plasmodium falciparum, Plasmodium ovale, Plasmodium malariae, and Plasmodium vivax. Among these, Plasmodium falciparum is responsible for over 90 % of malaria-related deaths worldwide. According to a 2019 report from the World Health Organization, there were over 200 million cases of malaria, leading to about half a million deaths globally, with children under the age of five being the most affected demographic. Malaria is endemic in >90 countries, making it a significant threat to global public health [1].

Over 90 % of malaria deaths occur in Africa. Children and pregnant women are particularly vulnerable to severe falciparum malaria; however, in regions with low endemicity and transmission, individuals of all ages are at risk due to low immunity [2].

An increased level of Plasmodium falciparum parasites in the bloodstream triggers an immune response, leading to the release of pro-inflammatory markers such as interleukin-10, tumor necrosis factor-alpha (TNF-alpha), and interferon-gamma. Plasmodium falciparum is known for its ability to adhere to various cells due to its erythrocyte membrane protein 1 (PfEMP1). This cytoadhesion allows the parasites to attach to the endothelium, platelets, and uninfected red blood cells, resulting in sequestration and obstruction of microcirculation. This interference impairs tissue perfusion and can lead to organ damage. Consequently, complications arise in the cerebral vasculature, manifesting as a clinicopathological syndrome known as cerebral malaria. Additionally, the rupture of parasitized small blood vessels can lead to subdural hemorrhage [1,3].

Treatment is administered using antimalarial medications that are tailored to the specific species involved and the resistance patterns observed in the relevant geographical regions. The effective management of this condition has been increasingly challenged by the growing resistance of Plasmodium falciparum to a range of therapeutic agents [4].

Common complications of falciparum malaria include cerebral malaria, renal failure, disseminated intravascular coagulation, and hepatic dysfunction [4]. Subdural hematoma, which refers to the collection of blood under the dura matter, has been reported in some adults with severe falciparum malaria [5]. An acute subdural hematoma typically occurs from the tearing of veins located between the arachnoid membranes and the dura mater. Head trauma is the most common cause of subdural hematomas, often resulting from motor vehicle accidents, falls, or assaults [6].

We present a case of an adult who developed recurrent spontaneous subdural hematomas while undergoing treatment for severe Plasmodium falciparum malaria. This condition is a very rare complication of malaria. We emphasize the importance of early imaging in patients experiencing neurological deficits due to falciparum malaria, as it helps to rule out subdural hematomas and enables prompt intervention to reduce the risk of mortality.

This case report has been reported in line with the SCARE criteria [7].

## 2 Case presentation

We present a case of a 40-year-old male who was admitted to our facility with a 2-day history of altered mental status and loss of consciousness on the day of admission. There was no trauma or seizures reported.

Four days before his admission, the patient was diagnosed with severe malaria and was undergoing treatment for it. He had experienced two previous episodes of malaria in the past two months, making this his third episode. During these malaria episodes, his main symptoms were headaches and generalized body malaise.

This was the patient's first admission. There is no history of chronic illnesses, and there is no personal or family history of coagulopathies. The patient has no history of smoking or alcohol consumption.

**Fig. 1:**
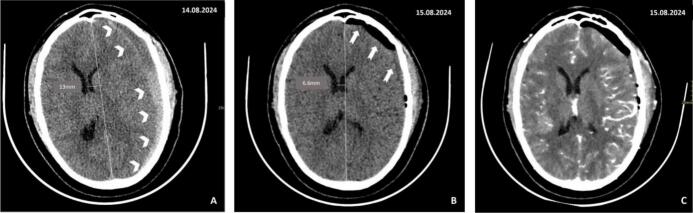
A. Baseline plain brain CT demonstrated acute subdural hemorrhage (arrowheads) with a midline shift of 13 mm (measured) B. Post burr hole and evacuation of subdural hematoma plain CT demonstrated a pocket of air at the left frontal subdural space (bolded arrows). A midline shift of 6.6 mm was also observed (measured) C. Cerebral angiography demonstrated normal findings.

During the general examination, no positive findings were noted. The neurological examination revealed that the patient had a Glasgow Coma Scale score of 12 (Eye – 3, Motor – 6, Verbal – 3). There were no signs of meningeal irritation or neurological deficits observed. The patient's vital signs were as follows: blood pressure of 145/84 mmHg, pulse rate of 62 beats per minute, respiratory rate of 14 breaths per minute, body temperature of 36.6 °C, and oxygen saturation of 98 % in room air. A CT scan of the head revealed an acute left subdural hematoma with mass effect and a 13 mm midline shift as seen in Fig. 1A.

Initial Investigations revealed a white blood cell count of 8.13 × 10^9^/L (reference range: 4–11 × 10^9^/L), with haemoglobin count of 11.9 g/dL (reference range: 11–16.5 g/dL), platelet count of 349 × 10^9^/L (reference range: 150–450 × 10^9^/L), a C-reactive protein concentration of 2.46 mg/dL (reference range < 5 mg/dL), Blood Urea Nitrogen of 4.6 mmol/L (reference range: 1.79–6.43 mmol/L), Serum creatinine of 51.36 umol/L (reference range: 59–104 umol/L), Total Serum bilirubin was 8.08 umol/L (reference range: 0–21 umol/L), Direct Serum Bilirubin was 4.48 umol/L (reference range: 0–5 umol/L), Serum SGOT/AST of 14.2 IU/L (reference range: 0–40 IU/L), Serum SGPT/ALT of 33.58 (reference range: 0–40 IU/L), Serum Gamma GT of 91.73 IU/L (reference range: 8–61 IU/L), Serum Alkaline Phosphatase of 50.98 IU/L (reference range: <250 IU/L), Serum Sodium of 133 mmol/L (reference range: 135–145 mmol/L), Serum Potassium of 4.11 mmol/L (reference range: 3.5–5 mmol/L), Serum Chloride of 95.78 mmol/L (reference range: 98–107 mmol/L), Prothrombin time of 12.8 s, Activated Partial thromboplastin time of 30.7 s, and International Normalized Ratio (INR) of 1.16 The rapid test for malaria antigen was positive for plasmodium falciparum with a parasite count of 50 trophozoites per 200 white blood cells. He had received a full dose of artesunate injections as treatment for malaria before his admission to our facility. Upon admission, he continued with a dose of four tablets of artemether-lumefantrine (40/480 mg strength) at 0 and 8 h on the first day, followed by 12-h intervals for the next two days as part of his ongoing malaria management.

Emergency surgical intervention was done whereby, two left frontoparietal and parietal occipital burr holes were drilled. There was liquified blood in the subdural space with membrane formation compressing the brain tissue, which was noted to be pulsating. All membranes were opened to expose the brain. About 50 cc of old blood was evacuated and irrigation with normal saline was done. After 24 h, a post-operative computed tomography (CT) scan of the head revealed that a significant hematoma had been evacuated. The scan showed a resolution of the mass effect with minimal evidence of midline shift. Additionally, a pocket of air was noted in the left frontal subdural space as seen in Fig. 1B.

**Fig. 2:**
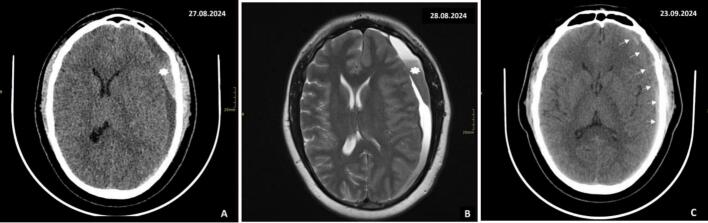
A. Plain CT of the brain, 2 weeks after initial treatment, demonstrated new mixed hyper and hypodense collections within the left subdural space (star) measuring 14.5 mm at the thickest point. It was causing a significant mass effect with a midline shift of about 9.2 mm. There was diffuse effacement of sulci at the left hemisphere. B. Plain MRI of the brain, 2 weeks after initial treatment, demonstrated left frontoparietal-temporal-occipital and temporal subdural collection (star) measuring 13 mm at the thickest point, causing mass effect onto the adjacent sulci with significant midline shift(6 mm). There was Minimal subfalcine herniation. C. Plain CT of the brain, 4 weeks after initial treatment, demonstrated a hyperdense subdural collection in the left subdural space (arrows) with a hypodensity in the non-dependent part measuring about 8.6 mm on the thickest part. There was a mass effect and an ipsilateral sulcal effacement with a mild midline shift of about 5 mm.

The patient was discharged after 72 h, and a Computed Tomography Angiography (CTA) was performed to rule out other vascular causes of the subdural hematoma, as shown in Fig. 1C. Two weeks after discharge, the patient returned with a severe throbbing headache. However, there were no accompanying symptoms such as nausea, vomiting, visual changes, seizures, or alterations in mental status. Upon suspicion of a recurrent subdural hemorrhage, a repeat CT scan was performed, revealing a subacute on chronic left subdural hemorrhage with a mass effect and a midline shift of 9.2 mm, along with left-sided cerebral edema as seen in Fig. 2A.

Magnetic resonance imaging of the brain revealed acute on chronic subdural hemorrhage, significant midline shift, subtle subfalcine herniation, and early onset hydrocephalus as shown in Fig. 2B.

The left burr holes were opened, and blood was evacuated, with simultaneous irrigation of previous burr holes and opening of the pseudo membrane. The brain was expanded completely to obliterate the space.

The patient received preoperative dexamethasone, starting with an initial dose of 8 mg every 8 h. Postoperatively, the dosage was tapered down to 6 mg every 8 h for two days. Additionally, the patient was administered an intravenous dose of tranexamic acid at 1000 mg as a STAT dose, followed by 500 mg every 8 h for 48 h. The patient was also prescribed atorvastatin at a dosage of 40 mg to be taken at night for 2 weeks. After completing this regimen, he was discharged and advised to continue follow-up as an outpatient.

## 3 Discussion

Subdural hematoma is defined as an accumulation of blood between the dura and arachnoid matter usually due to rupture of bridging veins following trauma or non-traumatic causes like coagulopathy, Arterial Venous Malformations (AVM) and ruptured aneurysm [8]. Recurrent subdural hematoma is the reaccumulating of ipsilateral hematoma following a surgical evacuation [9]. Malaria has been reported as a very rare cause of subdural hematoma and intracranial bleeds in a few cases around the world [10,11].

Malaria poses the greatest threat in tropical countries. As of 2022, around 96 % of global malaria related deaths occurred in 29 countries, with just four of these nations responsible for over half of the fatalities. Notably, Tanzania ranked as the fourth-highest contributor to this issue [2]. Our patient resides in Tanzania, which is a malaria-endemic country.

Malaria is diagnosed through the microscopic examination of stained blood smears, which aids in identifying specific species of the parasite. In this instance, Plasmodium falciparum was recognized. Additional diagnostic methods include rapid immunochromatographic tests for Plasmodium falciparum histidine-rich protein 2 and lactate dehydrogenase (LDH), as well as polymerase chain reaction (PCR) tests that detect parasite mRNA or DNA. Although PCR offers greater accuracy, it tends to be more costly and is often not readily available in many healthcare facilities in lower-income countries. The primary treatment for malaria involves administering antimalarial drugs, selected based on region-specific sensitivity patterns and the particular type of parasite identified [12,13]. Our patient was treated with intravenous Artesunate followed by oral artemether-lumefantrine as per the national guidelines for malaria treatment [12].

Malaria-induced coagulopathy, while rare, can disrupt the body's normal clotting mechanisms, leading to abnormal bleeding, including intracranial hemorrhage. In Plasmodium falciparum malaria, there is significant malaria-induced coagulopathy that leads to bleeding through mechanisms such as thrombocytopenia, platelet dysfunction, and impaired fibrinolysis [14].

Plasmodium falciparum-infected erythrocytes (PFIE) contribute to the upregulation of tissue factor (TF) expression on endothelial cells, which serve as crucial activators of the coagulation process. This phenomenon may occur through direct interaction with the infected cells or through the stimulation of tumor necrosis factor (TNF), a potent activator of TF [15].

PFIE also leads to the upregulation of adhesion molecules, such as von Willebrand factor (vWF), which plays a crucial role in platelet adhesion and aggregation. High levels of these molecules are observed in the early stages of the infection. Thrombocytopenia indicates an overconsumption or destruction of peripheral platelets [16].

Other key coagulation factors affected by the parasites include protein C, Protein S, and Antithrombin III. Studies show an inverse correlation between clinical severity and drastic improvement of these factors upon treatment [15,16]. These mechanisms contribute to bleeding tendencies that can complicate spontaneous subdural hematoma as seen in our patient.

There have been a few reported cases of spontaneous subdural hematoma as a complication of malaria. Huda et al. reported a case of a patient who presented with neurological symptoms 10 days after completing treatment for malaria and was subsequently diagnosed with a subdural hematoma, which is similar to our case. However, our patient developed symptoms on the fourth day of therapy for falciparum malaria [5]. Similarly, another case involved bilateral acute-on-chronic subdural hematoma, presenting with recurrent subdural hematoma and concurrent extradural and intraparenchymal bleeds [10]. While this case shared similarities with ours in terms of recurrent subdural hematoma, our patient did not develop intraparenchymal or extradural bleeds. Instead, the recurrence of subdural hematoma in our patient occurred after the resolution of falciparum malaria, likely due to the chronic inflammation (neomembrane) rather than malaria itself.

Meta-analyses have identified factors such as hematoma architecture, pneumocephalus, and significant midline shift as contributors to subdural hematoma recurrence. Oish et al. attributed recurrence to the presence of fragile capillaries in neomembrane formation. Similarly, our patient exhibited risk factors for recurrence, including a separated hematoma pattern with neomembrane, a.

13 mm midline shift, and pneumocephalus. Other known causes of recurrence, such as advanced age (over 75 years), liver disease, obesity, and anticoagulation therapy, were not present in our patient [[17], [18], [19]].

Some studies have reported a patient with concurrent infection with plasmodium falciparum and plasmodium vivax causing subdural hematoma and resistant to treatment [20]. However, our patient only had plasmodium falciparum detected which responded well to treatment.

The diagnosis of a subdural hematoma is typically confirmed with a CT scan of the head, which is regarded as the gold standard. An MRI of the brain can also be used to provide a more detailed characterization of the bleed, especially in cases of chronic subdural hematoma where neomembrane formation is present, aiding in preoperative planning. MRI is particularly sensitive to detecting smaller bleeds that may be difficult to identify on CT scans. Additionally, CT angiography is essential for ruling out other neovascular causes of subdural hematoma, such as arteriovenous malformations (AVM) and cerebral aneurysms. Both CT and MRI are valuable not only for diagnosing subdural hematomas but also for predicting their recurrence in certain cases [[17], [18], [19]]. Our patient underwent serial CT scans of the head to diagnose recurrence and monitor the response to surgical management of the subdural hematoma. Additionally, a CT angiography (CTA) was performed to rule out other vascular causes of the subdural hematoma.

Treatment options for subdural hematoma vary from conservative approaches to medical interventions, such as dexamethasone, atorvastatin, and tranexamic acid. The combination of surgery and pharmacotherapy has been shown to reduce recurrence rates [21,22]. Surgical interventions for hematoma treatment may include burr hole drainage, which is the standard procedure, as well as craniotomy or endoscopic evacuation [23]. Indications for surgery in subdural hematoma include a hematoma thickness >10 mm or a midline shift of >5 mm, as observed on a CT scan of the head, regardless of the patient's level of consciousness [24]. Our patient had a 12 mm midline shift, meeting the criteria for surgical intervention, and a 9.2 mm midline shift during the second presentation. Two burr holes were drilled in the left fronto-parietal and parieto-occipital regions for hematoma evacuation. A systematic review and meta-analysis by W. Liu et al., along with a randomized controlled trial by Sale D, demonstrated that postoperative drainage reduces the recurrence of subdural hematomas. However, they found that burr hole drainage or twist procedures do not significantly reduce recurrence rates or post-operative complications. While burr hole drainage is the standard procedure, a comparison between single and double burr holes revealed no statistical difference in outcomes, with recurrence rates of 3 % for single burr holes and 2.2 % for double burr holes [25,26]. The patient was scheduled for an outpatient follow-up clinic in which he progressed well with complete resolution of symptoms and hematoma.

## 4 Conclusions

Intracranial hemorrhagic complications, though rare in Plasmodium falciparum malaria, can present with nonspecific symptoms. Patients with altered consciousness or focal neurological signs should be evaluated for hemorrhagic complications, such as subdural hematoma. Early diagnosis and intervention can improve outcomes. While subdural hematoma is uncommon in malaria, physicians should maintain a high index of suspicion in endemic areas, especially in patients with a history of malaria and neurological symptoms.

## Author contribution

Irene Kilenzi was involved in the conception, acquisition, and interpretation of data, and drafting of the manuscript.

Jacqueline Gabone and Adil Datoo were involved in the interpretation of radiological data.

Elisamia Ngowi was involved in the literature review of the research work.

Mugisha Clement Mazoko supervised and reviewed the research work.

All authors read and approved the final manuscript.

## Ethical approval

Not required for case reports at our hospital for single case reports.

## Guarantor

Irene Kilenzi is the main guarantor of this research work. irenekilenzi@gmail.com

## Consent for publication

Written informed consent was obtained from the patient for publication of this case report and the accompanying images. A copy of the written consent is available for review by the corresponding author of this journal.

## Funding

No funds were needed to publish this case.

## Declaration of competing interest

All authors declare that there are no conflicts of interest.

## Data Availability

The datasets of the present study are available from the corresponding author upon request.

## References

[1] Zekar Lara ST. Plasmodium Falciparum Malaria. 2024 [cited September 2024]. StatPearls [Internet]: tatPearls Publishing, [cited September 2024]. Available from: https://www. ncbi.nlm.nih.gov/books/NBK555962/.

[2] Malaria eradication. Manila: WHO Regional Office for the Western Pacific.

[3] Milner D.A. (2018). Malaria pathogenesis. Cold Spring Harb. Perspect. Med..

[4] Garcia L.S. (2010). Malaria. Clin. Lab. Med..

[5] Huda M.F., Kamali N.I., Srivastava V.K., Kaif M. (2011). Spontaneous acute subdural hematoma in malaria: a case report. J. Vector Borne Dis..

[6] Ropper AH SM, Klein JP, Prasad S. Adams and Victor's Principles of Neurology. McGraw-Hill, New York2019.

[7] Sohrabi C., Mathew G., Maria N., Kerwan A., Franchi T., Agha R.A. (2023). The SCARE 2023 guideline: updating consensus surgical CAse REport (SCARE) guidelines. Int. J. Surg..

[8] Nouri A., Gondar R., Schaller K., Meling T. (2021). Chronic subdural hematoma (cSDH): a review of the current state of the art. Brain Spine..

[9] Ohba S., Kinoshita Y., Nakagawa T., Murakami H. (2013). The risk factors for recurrence of chronic subdural hematoma. Neurosurg. Rev..

[10] Zaidi S.M.F., Amjad A., Sohail K., Rehman F.U. (2024). A complex case of recurrent intracranial bleeds due to malaria-induced coagulopathy: a case report and literature review. Surg. Neurol. Int..

[11] Pallangyo P., Lyimo F., Nicholaus P., Kain U., Janabi M. (2016). Spontaneous subdural empyema following a high-Parasitemia <i>falciparum</i> infection in a 58-year-old female from a MalariaEndemic region. J. Investig. Med. High Impact Case Rep..

[12] Kileo N. Kileo N, editor: World Health Organization. 2017. [september 2024]. Available from: https://www.afro.who.int/news/monitoring-efficacy-antimalarial-medicines-tanzania#:∼:text=Therapeutic%20Efficacy%20Studies%20in%20Tanzania,using%20the%20standard% 20WHO%20protocol.

[13] Mishra S.K., Newton C.R.J.C. (2009). Diagnosis and management of the neurological complications of falciparum malaria. Nat. Rev. Neurol..

[14] Angchaisuksiri P. (2014). Coagulopathy in malaria. Thromb. Res..

[15] Francischetti I.M., Seydel K.B., Monteiro R.Q. (2008). Blood coagulation, inflammation, and malaria. Microcirculation.

[16] Moxon C.A., Heyderman R.S., Wassmer S.C. (2009). Dysregulation of coagulation in cerebral malaria. Mol. Biochem. Parasitol..

[17] Mishra R., Deora H., Florez-Perdomo W.A., Moscote-Salazar L.R., Garcia-Ballestas E., Rahman M.M. (2022). Clinical and radiological characteristics for recurrence of chronic subdural hematoma: a systematic review and Meta-analysis. Neurol. Int..

[18] Miah I.P., Tank Y., Rosendaal F.R., Peul W.C., Dammers R., Lingsma H.F. (2021). Radiological prognostic factors of chronic subdural hematoma recurrence: a systematic review and meta-analysis. Neuroradiology.

[19] Oishi M., Toyama M., Tamatani S., Kitazawa T., Saito M. (2001). Clinical factors of recurrent chronic subdural hematoma. Neurol. Med. Chir..

[20] Seshadri P., Dev A.V., Viggeswarpu S., Sathyendra S., Peter J.V. (2008). Acute pancreatitis and subdural haematoma in a patient with severe falciparum malaria: case report and review of literature. Malar. J..

[21] Scerrati A., Visani J., Ricciardi L., Dones F., Rustemi O., Cavallo M.A. (2020). To drill or not to drill, that is the question: nonsurgical treatment of chronic subdural hematoma in the elderly. A systematic review. Neurosurgical Focus..

[22] Qiu S., Zhuo W., Sun C., Su Z., Yan A., Shen L. (2017). Effects of atorvastatin on chronic subdural hematoma: a systematic review. Medicine (Baltimore).

[23] Bounajem M.T., Campbell R.A., Denorme F., Grandhi R. (2021). Paradigms in chronic subdural hematoma pathophysiology: current treatments and new directions. J. Trauma Acute Care Surg..

[24] Bullock MR, Chesnut R, Ghajar J, Gordon D, Hartl R, Newell DW, et al. Surgical management of acute subdural hematomas. Neurosurgery. 2006;58(3 Suppl):S16–24; discussion Si-iv. 16710968

[25] Liu W., Bakker N.A., Groen R.J.M. (2014). Chronic subdural hematoma: a systematic review and metaanalysis of surgical procedures. J. Neurosurg..

[26] Sale D. (2021). Single versus double Burr hole for drainage of chronic subdural hematoma: randomized controlled study. World Neurosurg..

